# Prognostic Association of Circulating Neutrophil Count with No-Reflow in Patients with ST-Segment Elevation Myocardial Infarction following Successful Primary Percutaneous Intervention

**DOI:** 10.1155/2017/8458492

**Published:** 2017-12-12

**Authors:** Jinfan Tian, Yue Liu, Yanfei Liu, Xiantao Song, Min Zhang, Feng Xu, Fei Yuan, Shuzheng Lyu

**Affiliations:** ^1^Department of Cardiology, Beijing Anzhen Hospital, Capital Medical University, Beijing 100029, China; ^2^Beijing Institute of Heart, Lung and Blood Vessel Diseases, Beijing 100029, China; ^3^Cardiovascular Disease Centre of Xiyuan Hospital, China Academy of Chinese Medical Sciences, Beijing 100091, China; ^4^Graduate School, Beijing University of Chinese Medicine, Beijing 100029, China

## Abstract

**Objective:**

The aim of the present study was to investigate the predictive value of neutrophil count for no-reflow in patients with ST-segment elevation myocardial infarction (STEMI) who underwent successful primary percutaneous intervention (PCI).

**Methods:**

We conducted a retrospective study of 361 patients diagnosed with acute STEMI between 2011 and 2015. All patients underwent successful PCI within 12 h from the onset of symptoms. Angiographic no-reflow was diagnosed based on a post-PCI thrombolysis in myocardial infarction flow grade ≤ 2 without mechanical obstruction. According to a neutrophil count cut-off determined by receiver operating characteristic curve analysis, patients were divided into two groups: group A (neutrophil count < 9.14 × 10^9^/L) and group B (neutrophil count ≥ 9.14 × 10^9^/L).

**Results:**

Compared to patients in the normal reflow group, patients with no-reflow had higher neutrophil counts (*P* < 0.05). The incidence rate of no-reflow in group A (18, 9.3%) was significantly lower than that in group B (38). Multivariate logistic regression analysis revealed that a neutrophil count ≥ 9.14 × 10^9^/L was independently predictive for no-reflow (odds ratio = 4.474, 95% confidence interval: 1.610–12.433, *P* = 0.004) after adjusting for potential confounders.

**Conclusions:**

A circulating neutrophil count ≥ 9.14 × 10^9^/L is independently associated with no-reflow in patients with acute STEMI following primary PCI.

## 1. Introduction

ST-segment elevation myocardial infarction (STEMI) is known to be one of the leading causes of mortality worldwide. Primary percutaneous coronary intervention (PCI) is the most effective way of preventing the progression of myocardial necrosis and reducing mortality associated with STEMI. However, according to Kloner et al. [1] under some circumstance, restoration of arterial flow into the previously ischemic tissue either does not occur or is greatly impeded. Early and adequate restoration of the infarct-related artery (IRA) does not always result in optimal myocardial reperfusion [2]. This phenomenon is defined as “no-reflow” [1, 2]. No-reflow reduces the benefits of primary PCI in patients with acute STEMI. The size of the “no-reflow” zone is closely correlated with cardiac systolic function, myocardial remodeling, ventricular arrhythmias, cardiogenic shock, mortality during hospitalization, and worsened outcomes at follow-up [3]. Currently, no single effective therapeutic approach is available for no-reflow, making prevention vital. Identifying patients at the greatest risk is the first step in the prevention of no-reflow [4]. It is necessary to detect available blood biomarkers and other clinical indices to predict the risk of no-reflow and reduce the incidence of this phenomenon in the early stage.

Myocardial ischemia/reperfusion injury is the most important pathological characteristic in the development of no-reflow [5]. Recent fundamental studies have shown that neutrophils become trapped in an area of myocardial ischemia reperfusion via the NF-*κ*B cascade [6–9]. Trapped leukocytes are established as important inflammation mediators of cardiac ischemia/reperfusion injury [8, 10–12]. Furthermore, clinical studies have reported that neutrophil accumulation at the coronary culprit lesion site predicts mortality in patients with acute coronary syndrome (ACS)/acute myocardial infarction (AMI) [13]. We hypothesized that the trapped neutrophils are derived from the circulating blood in the context of ischemia-reperfusion. With the above in mind, the aim of the present study was to investigate the prognostic association of an easily detectable biomarker—the count of circulating neutrophils with angiographic no-reflow assessed by post-PCI thrombolysis in myocardial infarction (TIMI) flow grade. Therefore, the neutrophil count on admission was considered in the present study.

## 2. Methods

### 2.1. Patients

A total of 361 patients diagnosed with acute STEMI from January 2011 to December 2015 were enrolled retrospectively in the present study. All patients underwent successful primary PCI within 12 hours from the onset of symptoms. The Ethics Committee of Anzhen Hospital approved the study protocol (Beijing, China).

### 2.2. STEMI Diagnostic Criteria

The STEMI diagnostic criteria are as follows: (1) typical ischemic chest pain lasting for at least 30 min and not alleviated by resting or nitroglycerin; (2) ST-segment elevation ≥ 2 mm in at least two consecutive leads or the onset of left bundle branch block; and (3) an increase and/or a decrease in cardiac biomarker values (preferably troponin), with at least one value above the 99th percentile of the upper reference limit [14].

### 2.3. Inclusion Criteria

The inclusion criteria are as follows: (1) patients diagnosed with STEMI; (2) men and nonpregnant women between 18 and 80 years of age; and (3) patients who signed the informed consent forms.

### 2.4. Exclusion Criteria

The exclusion criteria are as follows: (1) patients with cardiac shock; (2) patients with valvular heart disease; (3) patients with cardiomyopathy; (4) patients who underwent coronary artery bypass grafting; (5) heart transplant recipients; (6) patients with contraindications to antiplatelet agents and anticoagulation; (7) patients with multiple organ failure; (8) patients with acute infection, autoimmune disorders, or advanced cancer; and (9) patients allergic to contrast agents. All patients were divided into the no-reflow group and normal reflow group according to TIMI flow grade during coronary angiography (as illustrated below).

### 2.5. Diagnosis of No-Reflow during Coronary Angiography

All patients were administered with oral aspirin (300 mg) and clopidogrel (300 mg) and intravenous unfractionated heparin (50–70 U/kg). PCI procedures were performed via the transradial and transfemoral approaches. Before PCI, each patient underwent left and right coronary angiography with at least two projections. The upfront intracoronary administration of glycoprotein IIb/IIIa receptor inhibitor (GPIIb/IIIa receptor inhibitor, Tirofiban) was left to the operator's discretion during PCI. After intervention, all patients were administered with clopidogrel (75 mg) and aspirin (100 mg) once daily for 12 months. Other treatments were provided according to the physician's clinical opinion.

At least two experienced cardiologists determined all parameters and strategies. No-reflow was defined as a post-PCI TIMI flow grade of ≤2 in the IRA in the absence of dissection, spasm, apparent thrombus, or flow-limiting residual stenosis < 50%. TIMI flow grade 3 was considered as normal reflow [15–17].

### 2.6. Grouping

Based on the neutrophil count cut-off determined by the receiver operating characteristic (ROC) curve analysis, the patients were divided into two groups: group A (neutrophil count < 9.14 × 10^9^/L) and group B (neutrophil count ≥ 9.14 × 10^9^/L), namely, group A (<9.4 G/L) and group B (≥9.4 G/L).

### 2.7. Laboratory Analysis

Blood samples were drawn from the antecubital vein on admission for laboratory analysis. Neutrophil count was determined from the whole blood using an automated haematology analyser. Samples were centrifuged within 30 min to separate plasma and to determine the serum creatinine (Scr), blood glucose (GLU), and blood lipid profiles (low-density lipoprotein, high-density lipoprotein cholesterol, total cholesterol, and triglycerides) using an automated biochemical analyser. Cardiac biomarkers and high-sensitivity C-reactive protein (Hs-CRP) levels were measured using standard methods.

### 2.8. Clinical Data Collection and Quality Control

Responsible physicians performed the physical examinations and the independent researchers recorded data related to smoking history and comorbidities (diabetes mellitus and hypertension) on admission. The Killip classification was used to assess the severity of heart failure. Primary PCI strategy, choice of stent, and medications administered during hospitalization were chosen by the individual interventional cardiologists or responsible physicians according to clinical symptoms and angiographic characteristics. Data used for statistical analysis were obtained and entered into a computerized database by the staff.

### 2.9. Statistical Analysis

Continuous variables were presented as means ± standard deviations. When the variables were normally distributed, Student's *t*-test was used to compare two independent samples. The Mann–Whitney *U* test was used to compare nonnormally distributed data. Categorical variables were expressed as frequencies and percentages, and the chi-square test was used to compare the data. An ROC curve was used to determine neutrophil count cut-off level. The predictors of no-reflow were determined by univariate and multivariate logistic regression. In multivariate models, covariates included age ≥ 65, male, smoking history, hypertension, diabetes, Killip classification ≥ 3, the left anterior descending artery (LAD) as the IRA, neutrophil count ≥ 9.14 × 10^9^/L, neutrophil/lymphocyte ratio, cardiac troponin I (cTNI), upfront intracoronary GPIIb/IIIa receptor inhibitor administration, aspiration thrombectomy, platelet counts, white blood cell (WBC) counts, hemoglobin (HGB), time from symptom onset to reperfusion (>6 hours), multivessel disease, and initial TIMI flow grade (0-1) (those with a *P* value < 0.1 in univariate analysis and those that were clinically relevant). Results were presented as adjusted odds ratios (ORs) with 95% confidence intervals (CIs). A two-sided *P* value < 0.05 was considered statistically significant. SPSS 17.0 software was used to analyse the data.

## 3. Results

### 3.1. Baseline Characteristics

**Figure 1:**
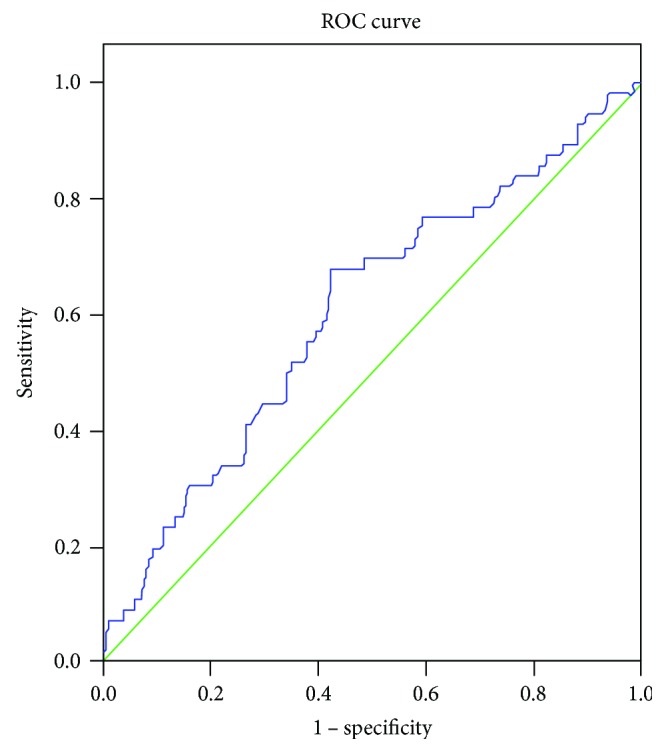
Receiver operating characteristic analysis of neutrophil count and no-reflow (area under the curve 0.604, 95% confidence interval: 0.522–0.687, *P* = 0.013).

ROC curve analysis revealed that neutrophil count predicted no-reflow. The area under the ROC curve was 0.604 (95% CI: 0.522–0.687, *P* = 0.013) (see Figure 1), and the neutrophil count cut-off value was 9.14 × 10^9^/L, with 67.9% sensitivity and 57.7% specificity. Patients were divided into two groups according to the neutrophil count cut-off level: group A (<9.4 G/L) (*n* = 194) and group B (≥9.4 G/L) (*n* = 167).

The mean age in group A (<9.4 G/L) was greater than that in group B (≥9.4 G/L). Differences in gender, smoking history, hypertension, diabetes, and history of PCI between the two groups were not statistically significant. No statistically significant differences were found between group A (<9.4 G/L) and group B (≥9.4 G/L) in terms of blood pressure, time from onset of symptoms to reperfusion, and multivessel disease. There were 53 (17.3%) patients with Killip class I in group A (<9.4 G/L) compared to 35 (21.1%) in group B (≥9.4 G/L). There were 128 (66.0%) patients with Killip class II in group A (<9.4 G/L) compared to 119 (71.7%) in group B (≥9.4 G/L). There were 11 (5.7%) patients with Killip class III in group A (<9.4 G/L) compared to eight (4.8%) in group B (≥9.4 G/L). There were 2 (1.0%) patients with Killip class IV in group A (<9.4 G/L) compared to four (2.4%) in group B (≥9.4 G/L). There were more patients with multistent implantation in group A (<9.4 G/L) (68, 35.1%) than in group B (≥9.4 G/L) (41, 24.6%). Higher WBC counts, neutrophil/lymphocyte ratios, red blood cell counts, HGB, platelet counts, and cTNI were detected in group B (≥9.4 G/L), whereas the lymphocyte counts and proportion of lymphocytes were decreased correspondingly. The differences in other blood markers were not statistically significant (see Table 1).

### 3.2. The Incidence of No-Reflow in Group A (<9.4 G/L) and Group B (≥9.4 G/L)

Eighteen (9.3%) patients in group A (<9.4 G/L) had no-reflow compared to 38 (22.8%) patients in group B (≥9.4 G/L). This difference was statistically significant (Table 2).

### 3.3. Clinical Characteristics of Patients in the Normal Reflow Group and No-Reflow Group

No differences between patients in the no-reflow group and normal reflow group were detected in terms of age, gender, smoking history, comorbidities, history of PCI, and blood pressure. Higher values of WBC counts, neutrophil counts, neutrophil proportions, and neutrophil to lymphocyte ratio were detected in the no-reflow group compared to those in the normal reflow group. Lymphocyte counts and the proportion of lymphocytes were lower in the no-reflow group than those in the normal reflow group. Blood lipids, GLU, and other blood markers were not statistically significantly different between the two groups. The differences between the two groups with respect to left ventricular diastolic dysfunction and ejection fraction on admission were not statistically significant. Patients with no-reflow had significantly higher Killip classifications than those with normal reflow (*P* < 0.05) (Table 3).

### 3.4. Coronary Angiography Findings and Percutaneous Intervention Characteristics in the No-Reflow and Normal Reflow Groups

No-reflow, defined according to TIMI flow grade during coronary angiography, was more frequently observed among patients with the LAD as the IRA, while TIMI flow grade 3 was more frequently observed within the right coronary artery (RCA). There were more patients with anterior wall infarction in the no-reflow group than in the normal reflow group. The upfront intracoronary GPIIb/IIIa receptor inhibitor use rate was lower in the no-reflow group than the normal flow group. However, the incidence of no-reflow was not affected by multivessel disease, multistent implantation, and aspiration thrombectomy. Moreover, there were no significant differences between the no-reflow and normal reflow groups with respect to time from symptom onset to reperfusion and initial TIMI flow grade. Non-IRA intervention was not associated with no-reflow (Table 4).

### 3.5. Univariate and Multivariate Logistic Regression

In univariate analysis, the Killip classification ≥ 3 (OR = 2.824, 95% CI: 1.155–6.904, *P* = 0.023), LAD as the IRA (OR = 1.821, 95% CI: 1.018–3.259, *P* = 0.043), neutrophil count ≥ 9.14 × 10^9^/L (OR = 2.880, 95% CI: 1.573–5.275, *P* = 0.001), neutrophil/lymphocyte ratio (OR = 1.067, 95% CI: 1.015–1.121, *P* = 0.011), cTNI (OR = 1.004, 95% CI: 1.000–1.008, *P* = 0.036), and WBC count (OR = 1.086, 95% CI: 1.002–1.178, *P* = 0.046) were predictors for no-reflow. Upfront intracoronary GPIIb/IIIa receptor inhibitor use was negatively associated with no-reflow (OR = 0.303, 95% CI: 0.091–1.010, *P* = 0.052). In multivariate logistic regression analysis, neutrophil count ≥ 9.14 × 10^9^/L was a predictor for no-reflow after adjusting for patients aged ≥ 65, male, smoking, hypertension, diabetes, Killip classification ≥ 3, LAD as the IRA, neutrophil/lymphocyte ratio, cTNI, upfront intracoronary GPIIb/IIIa receptor inhibitor use, aspiration thrombectomy, platelet counts, WBC counts, HGB, time from symptom onset to reperfusion (>6 hours), multivessel disease, and initial TIMI flow grade (0-1) (OR = 4.474, 95% CI: 1.610–12.433, *P* = 0.004) (Table 5).

## 4. Discussion

In the present study, there were 56 (15.5%) patients with angiographic no-reflow. Patients in the no-reflow group had significantly higher neutrophil counts than those in the normal reflow group. In univariate and multivariate logistic regression analyses, a neutrophil count above 9.14 × 10^9^/L was independently associated with no-reflow after adjusting for age ≥ 65, male, smoking history, hypertension, diabetes, Killip classification ≥ 3, LAD as the IRA, neutrophil/lymphocyte ratio, cTNI, upfront intracoronary GPIIb/IIIa inhibitor administration, aspiration thrombectomy, platelet counts, WBC counts, HGB, time from symptom onset to reperfusion (>6 hours), multivessel disease, and initial TIMI flow grade (0-1).

The present result is supported by the retrospective study of Kosuge et al. [18] stating that a WBC count of 12,000 cells/mm^3^ was an independent predictor of impaired myocardial reperfusion in patients with early recanalized anterior acute myocardial infarction (AMI). However, they only recruited the patients with anterior AMI. Furthermore, they did not analyse specific types of WBCs. Takahashi et al. [19] concluded that neutrophils over 10 G/L were associated with no reflow within 116 patients with a first anterior AMI. The present study recruited patients not limited to those with anterior AMI. The study of Wang et al. [20] which assessed the relationship between neutrophil counts and no-reflow included 217 patients in the author's center, age and blood cells counts were adjusted exclusively in the study, and the threshold of neutrophil count no-reflow was not analysed. In the present study, we obtained a cut-off value of 9.14 × 10^9^/L (67.9% sensitivity and 57.7% specificity), with neutrophil counts above this threshold being associated with increased rates of no-reflow. There were studies on the prognostic value of neutrophil to lymphocyte ratio in predicting no-reflow [15, 16]; hence, the present study also included neutrophil/lymphocyte ratio in the multivariate analysis. Additionally, other factors that are clinically relevant to no-reflow including hypertension, blood pressure, Killip classification (≧3), cTNI, time from symptom onset to reperfusion (>6 hours), multivessel disease, and initial TIMI flow grade (0-1) were also included in the multivariate analysis in the present study. The prognostic association of the neutrophil/lymphocyte ratio with no-reflow was lost after adjusting for neutrophil count ≥ 9.14 × 10^9^/L. Furthermore, neutrophil/lymphocyte ratio was not associated with no-reflow in multivariate analysis without confounding for neutrophil counts (OR = 1.043, 95%CI: 0.984–1.106, *P* = 0.152). This could be explained by the elevated neutrophil/lymphocyte ratio in our study that mainly resulted from the increased neutrophil counts.

The underlying mechanism of the involvement of neutrophils in no-reflow is complex. Ischemic injury damages myocardiocytes, which presents as myocardial cell swelling and interstitial edema. The pathological changes in myocardial cells increase the compression of intramural vessels and induce neutrophil plugging and activation in the coronary microcirculation [4]. The oxygen-free radicals released by activated neutrophils contribute to endothelial injury and impaired reperfusion. At the time of reperfusion, there was a massive neutrophil adhesion to the endothelium due to the excessive generation of reactive oxygen species and subsequently activated NF-*κ*B cascade. Structural luminal obstruction of the microvasculature resulted from microaggregates formed by neutrophils and platelets that aggravate the reperfusion injury [4, 21]. Moreover, intense and prolonged coronary microvascular vasoconstriction is attributable to vasoactive substances produced by neutrophils, platelets, and damaged endothelial cells [11]. In addition, the infiltration of neutrophils in the vulnerable myocardium as a result of increased vascular permeability enhances interstitial edema and extravascular mechanical compression, contributing to the pathological processes of no-reflow [4, 11].

ACS is a group of clinical syndromes characterized by rupture or erosion of coronary atherosclerotic plaques and subsequent complete or incomplete thrombotic occlusion [22]. Lipid-rich plaques are correlated with impaired reperfusion after restoration of the epicardial artery [23]. Noncalcified plaque burden is correlated with the neutrophil/lymphocyte ratio [24]. Neutrophils mediate apoptosis in endothelial cells and smooth muscle cells, contributing to plaque rupture [25–27]. Microvascular embolization and no-reflow occur when a mass of plaque fragments, leading to the release of cholesterol crystals and microthrombi into the bloodstream. Moreover, active neutrophils accelerate the formation of platelet-leukocyte aggregates, plugging the microvessel.

In the present study, we found that the Killip classification ≥ 3 was associated with no-reflow. Patients with no-reflow had advanced Killip classifications, coincident with the findings of Zhou et al. [17], despite that Killip classification 2 rate in the present was higher than that reported. There were 7.9% patients with Killip classifications 3 and 4, similar to the percentage in the study of Zhou et al. (8.9%) [17]. Killip classification of at least grade 3 on admission may be associated with larger infarctions and decreased coronary perfusion pressure [17]. The decreased coronary pressure accelerates plugging of neutrophils in the microvasculature, inducing no-reflow. Although we observed the cTNI (associated with larger infarct area) level was higher in group B (<9.4 G/L) than in group A (≥9.4 G/L), there was no difference between the no-reflow group and normal reflow group. Furthermore, after adjusting for cTNI, neutrophil count was independently associated with no-reflow.

### 4.1. Limitations

There are some limitations to the present study. A relatively small sample size was used. Furthermore, only TIMI flow grade was used to identify no-reflow, and no other diagnostic methods were used because of limited data. This partially explains why the LAD as the IRA was a negative factor for no-reflow. However, this difference did not affect the outcomes of the study. A prospective study including a larger sample, and in which TIMI myocardial perfusion or myocardial blush grade is assessed, is needed in the future.

## 5. Conclusions

Inflammatory responses and the infiltration of neutrophils are associated with ischemia/reperfusion injury associated with no-reflow following PCI. A circulating neutrophil count ≥ 9.14 × 10^9^/L is independently associated with no-reflow in patients with acute STEMI following successful primary PCI.

## Figures and Tables

**Table 1:** Baseline characteristics in group A and group B.

Parameters	Group A (<9.4 G/L) (*n* = 194)	Group B (≥9.4 G/L) (*n* = 167)	*P* value
Age (years, $\bar{X}\pm S$)	56.48 ± 10.85	53.57 ± 10.86	0.012^∗^
Gender, male, *n* (%)	171 (88.1)	148 (88.6)	0.888
Smoker, *n* (%)	129 (66.5)	120 (71.9)	0.272
Hypertension, *n* (%)	105 (54.1)	90 (53.9)	0.965
Diabetes, *n* (%)	50 (25.8)	44 (26.3)	0.901
History of PCI, *n* (%)	12 (6.2)	7 (4.2)	0.398
SBP (mmHg, $\bar{X}\pm S$)	120.12 ± 17.93	116.60 ± 17.11	0.074
DBP (mmHg, $\bar{X}\pm S$)	74.93 ± 10.94	73.82 ± 11.40	0.579
Time from onset of symptoms to reperfusion (hour)	5.19 ± 2.72	5.25 ± 2.44	0.443
Killip classification, *n* (%)			0.395
1	53 (17.3)	35 (21.1)	
2	128 (66.0)	119 (71.7)	
3	11 (5.7)	8 (4.8)	
4	2 (1.0)	4 (2.4)	
WBC counts (×10^9^/L)	8.83 ± 1.69	13.83 ± 2.62	0.0001^∗∗^
Lymphocyte counts (×10^9^/L)	1.65 ± 0.89	1.46 ± 0.81	0.014^∗^
Proportion of lymphocytes (%)	18.84 ± 9.04	10.70 ± 5.25	0.0001^∗∗^
Neutrophil/lymphocyte ratio	5.38 ± 4.12	9.89 ± 4.88	0.0001^∗∗^
Red blood cell counts (×10^12^/L)	4.56 ± 0.48	4.76 ± 0.57	0.0001^∗∗^
HGB (g/L)	141.45 ± 14.33	145.72 ± 14.84	0.006^∗∗^
Platelet counts (×10^9^/L)	202.65 ± 55.48	221.62 ± 71.07	0.001^∗∗^
PDW (%)	12.15 ± 1.85	12.46 ± 1.82	0.115
cTNI (ng/L)	65.64 ± 55.83	90.63 ± 71.91	0.0001^∗∗^
Scr (*μ*mol/L)	76.99 ± 18.63	79.37 ± 23.36	0.489
TG (mmol/L)	1.86 ± 1.50	1.84 ± 1.01	0.419
TCHO (mmol/L)	4.62 ± 0.89	4.65 ± 0.98	0.811
HDL-C (mmol/L)	1.02 ± 0.23	0.99 ± 0.23	0.246
LDL-C (mmol/L)	2.91 ± 0.78	3.02 ± 0.87	0.245
GLU (mmol/L)	7.88 ± 2.59	8.43 ± 3.07	0.129
Hs-CRP (mg/L)	9.54 ± 10.14	11.75 ± 10.47	0.051
FBG (g/L)	2.72 ± 0.56	2.72 ± 0.71	0.985
IRA, *n* (%)			0.802
LAD	95 (49.0)	79 (47.3)	
LCX	18 (9.3)	19 (11.4)	
RCA	81 (41.8)	69 (41.3)	
Multivessel disease, *n* (%)	48 (24.7)	42 (25.1)	0.929
Multistent, *n* (%)	68 (35.1)	41 (24.6)	0.030^∗^

^∗^ *P* < 0.05; ^∗∗^*P* < 0.01. SBP: systolic blood pressure; DBP: diastolic blood pressure; WBC: white blood cell; HGB: hemoglobin; PDW: platelet distribution width; cTNI: cardiac troponin I; Scr: serum creatinine; TG: triglyceride; TCHO: total cholesterol; HDL-C: high-density lipoprotein cholesterol; LDL-C: low-density lipoprotein cholesterol; GLU: glucose; Hs-CRP: high-sensitivity C-reactive protein; FBG: fibrinogen; IRA: infarct-related artery; LAD: left anterior descending; LCX: left circumflex artery; RCA: right coronary artery.

**Table 2:** The incidence of no-reflow in group A and group B.

Parameters	Group A (<9.4 G/L) (*n* = 194)	Group B (≥9.4 G/L) (*n* = 167)	*P* value
No-reflow, *n* (%)			0.0001^∗∗^
Yes	18 (9.3)	38 (22.8)	
No	176 (90.7)	129 (77.2)	

^∗∗^ *P* < 0.01.

**Table 3:** Clinical characteristics of patients with normal reflow and no-reflow.

Parameters	Normal reflow (*n* = 305)	No-reflow (*n* = 56)	*P* value
Age (years, $\bar{X}$ ± S)	54.96 ± 11.80	56.11 ± 11.66	0.470
Gender, male, *n* (%)	269 (88.2)	50 (89.3)	0.815
Smoker, *n* (%)	212 (69.5)	37 (66.1)	0.609
Hypertension, *n* (%)	165 (54.1)	30 (53.6)	0.942
Diabetes, *n* (%)	80 (26.2)	14 (25.0)	0.847
History of PCI, *n* (%)	18 (5.9)	1 (1.8)	0.330
SBP (mmHg, $\bar{X}$ ± S)	118.8 ± 17.6	116.9 ± 17.7	0.504
DBP (mmHg, $\bar{X}$ ± S)	74.4 ± 11.3	74.6 ± 10.6	0.983
Killip classification, *n* (%)			0.011^∗^
1	82 (26.9)	7 (12.5)	
2	206 (67.5)	41 (73.2)	
3	12 (3.9)	7 (12.5)	
4	5 (1.6)	1 (1.8)	
WBC counts (×10^9^/L)	10.99 ± 3.23	11.97 ± 3.61	0.042^∗^
Proportion of neutrophils (%)	78.73 ± 11.11	82.73 ± 9.15	0.002^∗∗^
Neutrophil counts (×10^9^/L)	8.79 ± 3.21	10.01 ± 3.45	0.013^∗^
Lymphocyte counts (×10^9^/L)	1.61 ± 0.89	1.34 ± 0.45	0.034^∗^
Proportion of lymphocytes (%)	15.59 ± 8.69	12.29 ± 7.19	0.002^∗∗^
Neutrophil/lymphocyte ratio	7.17 ± 4.93	9.10 ± 5.19	0.001^∗∗^
Red blood cell counts (×10^12^/L)	4.66 ± 0.53	4.62 ± 0.56	0.649
HGB (g/L)	143.71 ± 14.55	141.88 ± 15.54	0.391
Platelet counts (×10^9^/L)	212.57 ± 64.85	205.19 ± 57.79	0.467
PDW (%)	12.27 ± 1.87	12.44 ± 1.70	0.529
cTNI (ng/L)	74.09 ± 62.49	94.15 ± 75.05	0.078
Scr (μmol/L)	77.66 ± 21.19	80.45 ± 19.61	0.155
TG (mmol/L)	1.88 ± 1.37	1.70 ± 0.83	0.795
TCHO (mmol/L)	4.65 ± 0.92	4.53 ± 1.00	0.382
HDL-C (mmol/L)	1.00 ± 0.23	1.04 ± 0.25	0.310
LDL-C (mmol/L)	2.97 ± 0.81	2.92 ± 0.90	0.696
GLU (mmol/L)	8.17 ± 2.94	7.95 ± 2.19	0.717
Hs-CRP (mg/L)	10.62 ± 10.48	10.21 ± 9.49	0.869
FBG (g/L)	2.73 ± 0.64	2.64 ± 0.63	0.574
LVDD (mm)	49.70 ± 4.65	49.09 ± 7.81	0.728
EF (%)	54.30 ± 9.49	52.91 ± 10.28	0.355

^∗^ *P* < 0.05; ^∗∗^*P* < 0.01. LVDD: left ventricular diastolic diameter; EF: ejection fraction.

**Table 4:** Percutaneous intervention findings of patients with normal reflow and no-reflow.

Parameters	Normal reflow (*n* = 305)	No-reflow (*n* = 56)	*P* value
Time from onset of symptoms to reperfusion (hour)	5.20 ± 2.70	5.29 ± 1.95	0.315
Time from onset of symptoms to reperfusion (>6 hours)	96 (31.5)	16 (28.6)	0.666
Multivessel disease, *n* (%)			
Yes	76 (24.9)	14 (25.0)	0.990
No	229 (75.1)	76 (24.9)	
Initial TIMI flow grade, *n* (%)			
0-1	192 (63)	38 (67.9)	0.483
≥2	113 (37.1)	18 (32.1)	
Multistent, *n* (%)			
Yes	93 (30.5)	16 (28.6)	0.774
No	212 (69.5)	40 (71.4)	
IRA, *n* (%)			
LAD	140 (45.9)	34 (60.7)	0.051
LCX	30 (9.8)	7 (12.5)	
RCA	135 (44.3)	15 (26.8)	
Infarct location, *n* (%)			0.149
Anterior wall	140 (45.9)	36 (64.3)	
Inferior wall	55 (18)	6 (10.7)	
Inferior and posterior wall	43 (14.1)	4 (7.1)	
Complicate by right ventricular	63 (20.7)	10 (17.9)	
Others	4 (1.3)	0 (0)	
Aspiration thrombectomy			
Yes	206 (67.5)	40 (71.4)	0.566
No	99 (32.5)	16 (28.6)	
Upfront intracoronary GPIIb/IIIa receptor inhibitor			
Yes	48 (15.7)	3 (5.4)	0.040^∗^
No	257 (84.3)	53 (94.6)	
Non-IRA intervention, *n* (%)			
Yes	18 (5.9)	4 (7.1)	0.760
No	287 (94.1)	52 (92.9)	

^∗^ *P* < 0.05.

**Table 5:** Univariate and multivariate logistic analysis for no-reflow.

Parameters	Univariate logistic analysis			Multivariate logistic analysis		
	OR	95% CI	*P*	OR	95%CI	*P*
Age ≥ 65 years	1.116	0.567–2.199	0.751	1.068	0.467–2.442	0.877
Male	1.115	0.446–2.786	0.815	1.364	0.441–4.224	0.590
Smoking	1.171	0.640–2.143	0.610	1.148	0.549–2.400	0.714
Hypertension	1.021	0.577–1.809	0.942	0.979	0.519–1.845	0.947
Diabetes	0.938	0.486–1.807	0.847	0.821	0.395–1.710	0.599
Killip classification (≧3)	2.824	1.155–6.904	0.023^∗^	4.072	1.391–11.916	0.010^∗^
LAD as IRA	1.821	1.018–3.259	0.043^∗^	2.457	1.226–4.925	0.011^∗^
Neutrophil count ≥ 9.14 × 10^9^/L	2.880	1.573–5.275	0.001^∗∗^	4.474	1.610–12.433	0.004^∗∗^
Neutrophil/lymphocyte ratio	1.067	1.015–1.121	0.011^∗^	1.029	0.967–1.095	0.366
cTNI	1.004	1.000–1.008	0.036^∗^	1.001	0.995–1.006	0.806
Upfront intracoronary GPIIb/IIIa receptor inhibitor	0.303	0.091–1.010	0.052	0.219	0.061–0.783	0.019^∗^
Aspiration thrombectomy	1.201	0.642–2.250	0.566	1.253	0.469–3.347	0.652
Platelet counts	0.998	0.993–1.003	0.424	0.997	0.991–1.003	0.343
WBC counts (×10^9^/L)	1.086	1.002–1.178	0.046^∗^	0.940	0.798–1.107	0.459
HGB (g/L)	0.992	0.973–1.011	0.390	0.982	0.959–1.007	0.159
Time from symptoms onset to reperfusion (>6 hours)	0.871	0.465–1.632	0.666	0.932	0.469–1.851	0.840
Multivessel disease	1.004	0.520–1.940	0.990	0.987	0.470–2.075	0.973
Initial TIMI flow grade (0-1)	1.242	0.677–2.280	0.483	0.886	0.354–2.219	0.796

^∗^ *P* < 0.05; ^∗∗^*P* < 0.01. Age ≥ 65 years, male, smoking, hypertension, diabetes, Killip classification (≧3), LAD as IRA, neutrophil count ≥ 9.14 × 10^9^/L, neutrophil/lymphocyte ratio, cTNI, upfront intracoronary GPIIb/IIIa receptor inhibitor, aspiration thrombectomy, platelet counts, WBC counts, HGB, time from symptoms onset to reperfusion (>6 hours), multivessel disease, and initial TIMI flow grade (0-1) were included in the multivariate analysis.
